# Investigation of Diabetic Kidney Disease in the Network for Pancreatic Organ Donors with Diabetes-Kidney Cohort

**DOI:** 10.34067/KID.0000000664

**Published:** 2025-01-30

**Authors:** Gabriel Lerner

**Affiliations:** Yale University School of Medicine, New Haven, Connecticut

**Keywords:** diabetes, histopathology, renal pathology

The prevalence of diabetes is increasing in the United States, now believed to be present in almost one in six adults in the United States (15.8%^1^). Diabetic nephropathy (DN) and the development of diabetic kidney disease (DKD) are major complications in patients with diabetes.^2^ Prevention has long been of clinical interest because the development of DKD is associated with increased mortality in those with both type 1 and type 2 diabetes.^3,4^ Pathologically, DN is characterized by glomerular mesangial expansion (including the periodic acid–Schiff [PAS] positive nodular Kimmelstiel–Wilson nodules of class 3 DN) and glomerulosclerosis, glomerular and tubular basement membrane thickening, arteriolar hyalinosis, and progressive tubulointerstitial inflammation, fibrosis, and atrophy.^5^ Little, however, is known of the precise mechanisms of progression of DKD. Limited access to human DKD biopsy tissue and lack of suitable animal models encompassing the entirety of disease progression have complicated systematic evaluation of this disease.^6^

The Network for Pancreatic Organ Donors with Diabetes (nPOD) cohort is a collaborative research initiative (https://npod.org) which collects and provides access to diabetes-related human tissues from organ donors with long-standing diabetes.^7^ Previous work by the consortium has focused on use of pancreatic tissues. The article by Ward *et al.*, presented in this issue of the *Kidney360*, instead presents an investigation and analysis of kidney tissues from nPOD cohort donors, analyzed using both traditional morphologic and deep learning-based approaches.^8^ This investigative work defines a potentially valuable tissue cohort for future investigators interested in the study of mechanisms of DKD and those interested in preclinical validation of possible biomarkers or therapeutic targets.

Ward *et al.*, in collaboration with nPOD investigators, prospectively identified 48 donor kidneys for study inclusion from organ donors with either type 1 or type 2 diabetes or no DKD. Additional inclusion criteria included diabetes >8 years' duration, and an eGFR meeting criteria for CKD stages 3 or 4. Kidney tissue was obtained in formalin fixed paraffin embedded blocks for immunofluorescence studies, with additional material snap-frozen and embedded in optimal cutting temperature media for future use. The authors stratify their cohort by both histologic DN class and the presence or absence of a clinical diagnosis of diabetes. Analyses included both traditional morphologic assessment by pathologists according to standard diagnostic criteria,^5^ as well as automated computational pathology approaches.

Cases were stained with hematoxylin and eosin, PAS, and picrosirius red, and were digitally scanned to allow for computational analysis. Semiquantitative pathologist-assigned DN histologic scores were relatively concordant with quantitative automated computational assessment of the same features, including mesangial expansion (*κ* 0.65; see Figure 2, B and C)^8^ and tubular atrophy (*κ* 0.77; see Figure 2, E and F).^8^ Terminal deoxynucleotidyl transferase–mediated digoxigenin-deoxyuridine nick-end labeling staining was performed to evaluate for the extent of apoptosis as an indirect measurement of postmortem tissue integrity. There was no significant different in the percent terminal deoxynucleotidyl transferase–mediated digoxigenin-deoxyuridine nick-end labeling-positive cells across groups, indicating viable tissue for further study (see Figure 2, I and J).^8^

The authors emphasize that all histologic classes of DN (classes 1–4) were observed in whole-slide images of individual cases scored as class 3 DN. They call attention to the potential for inaccurate staging or sampling bias that exists in the small tissue samples obtained during routine clinical practice.

Owing to the large size of each cortical section (containing up to 50,000 tubular profiles and 900 glomeruli, per the authors' analysis), use of a computational approach was necessary to provide accurate quantitative assessment of diagnostic features. Training of the computational pipeline involved first aligning scanned epifluorescent and PAS-stained whole-slide images from a diverse set of training cases from each DN Class (see Supplemental Figure 1).^8^ Cortical and medullary areas were manually delineated, as were glomeruli, large vessels, and tubulointerstitium. Glomerular and tubular detection applications were trained and then tested and validated with separate images before use with scanned study biopsy images (see Supplemental Figure 1).^8^

The authors performed *in silico* reanalysis of a previously published bulk RNA transcriptomic DKD dataset.^9^ This unbiased approach allowed for examination and confirmation of expected molecular changes in the nPOD cohort. Markers identified by this approach included *PTPRO* and *CORO2B* (both expressed in healthy podocytes), as differentially expressed in DKD glomeruli. Evaluation of nPOD samples showed downregulation of both markers in DKD class 3 podocytes relative to no DKD controls (see Figure 3, E–H).^8^ PROM1, a marker of cellular proliferation, was upregulated in *in silico* DKD transcriptional analysis. The authors showed relatively low PROM1 expression by immunofluorescence in no DKD cases, with highest PROM1 expression in injured tubules in DKD cases (see Figure 5, C and D),^8^ indicating increased cell turnover relative to no DKD controls (either with or without diabetes).

Traditional cell type-specific markers were also evaluated across these cases. The podocyte marker WT1 (see Figure 3, A and B)^8^ and endothelial marker CD31 (see Figure 3, C and D),^8^ both showed expected statistically significant reductions in class 3 DKD cases, relative to controls. *Lotus tetragonolobus* was examined as a marker of intact proximal tubular brush borders (see Figure 4, A and B),^8^ and the sodium-chloride cotransporter as a marker of the first and second segments of the distal convoluted tubular compartment (see Figure 4, C and D).^8^ *Dolichos biflorus agglutinin* was evaluated as a marker of collecting ducts (see Figure 4, E and F).^8^ Of these markers, *Lotus tetragonolobus* and sodium-chloride cotransporter showed decreased expression in class 3 DKD cases relative to controls, indicative of increasing proximal tubular and collecting duct injury, respectively. Dolichos biflorus agglutinin showed no significant difference across case types. The prognostic tubular injury marker kidney injury molecular 1 also showed a statistically significant increase among DKD class 3 cases relative to controls (see Figure 5, A and B).^8^

Ward *et al.*’s rigorous investigation into the expression patterns of these cell type-specific markers in the nPOD DKD sections reveal results that might be expected on the basis of our knowledge of DKD pathophysiology. The strength of their analysis, beyond tissue-based validation of targets identified from an *in silico* approach, is that it serves to validate the integrity and suitability of the nPOD-Kidney tissue cohort for further research efforts.

One wonders whether, given large enough case numbers, expression could be probed across specific DN classes (*e.g*., class 1, 2A, 2B, or 3). It is likely that the currently accepted DN histopathologic classification system on the basis of degree of mesangial matrix deposition oversimplifies a heterogeneous disease process. Could applying a multiplex panel of markers for known or as of yet unknown biomarkers, discovered through transcriptomic evaluations such as those undertaken here, provide prognostic or predictive information beyond current morphologic classification? Indeed, although a daunting prospect, such efforts are well underway.^10^

The authors should be commended on the developing and validating the nPOD-Kidney cohort as clinicopathologic resource for use by future investigators in DKD. Such tissue will provide a robust resource for those interested in examining biomarkers discovered in preclinical or animal models, before investigation in clinical trials. The authors' use of automated computational algorithms to quantify clinically relevant pathologic features in kidney tissues is also commendable. Such approaches are likely to become a part of routine pathology practice in the coming years. These technologies are not likely to replace the key diagnostic role of nephropathologists, but may in the future fill a valuable supplemental diagnostic role in the pathology workflow.^11^

## Supplementary Material
