# Supplementary material for: Investigation of Diabetic Kidney Disease in the Network for Pancreatic Organ Donors with Diabetes-Kidney Cohort
Source: Kidney360. 2025 Jan 30;6(1):1–3. doi: 10.34067/KID.0000000664 (PMC11793179; doi:10.34067/KID.0000000664)
Supplement: Supplementary file 1 [file kidney360-6-001-s001.pdf]

ASN American Society of Nephrology · JASN Journal of the American Society of Nephrology · KIDNEY360 Accessing Our World From Every Angle · CJASN Clinical Journal of the American Society of Nephrology

ASN Journal Disclosure Form

As per ASN journal policy, I have disclosed any financial relationships or commitments I have held in the past 36 months as included below. I have listed my Current Employer below to indicate there is a relationship requiring disclosure. If no relationship exists, my Current Employer is not listed.

G. Lerner has nothing to disclose.

I understand that the information above will be published within the journal article, if accepted, and that failure to comply and/or to accurately and completely report the potential financial conflicts of interest could lead to the following: 1) Prior to publication, article rejection, or 2) Post-publication, sanctions ranging from, but not limited to, issuing a correction, reporting the inaccurate information to the authors' institution, banning authors from submitting work to ASN journals for varying lengths of time, and/or retraction of the published work.

Name: Gabriel B. Lerner
Manuscript ID: K360-2024-000996
Manuscript Title: Editorial Commentary on: 'nPOD-Kidney: A Heterogenous Donor Cohort for the Investigation of Diabetic Kidney Disease Pathogenesis and Progression'
Date of Completion: November 15, 2024
Disclosure Updated Date: May 22, 2024
